# The Mortality of Older Adult Inpatients with Kidney Cancer during the Pandemic: A Retrospective Cohort Study

**DOI:** 10.1093/geroni/igab046.3745

**Published:** 2021-12-17

**Authors:** Kendall Brune, Cheng Yin, Liam O'Neill, Rongfang Zhan

**Affiliations:** 1 Meharry Medical College, Nashville, Tennessee, United States; 2 University of North Texas, Corinth, Texas, United States; 3 University of North Texas, University of North Texas, Texas, United States

## Abstract

Background: Elderly patients are a vulnerable group during the Covid-19 pandemic, especially those with cancer. Our study aims to identify how Covid-19 impacts elderly inpatients with kidney cancer and determine risk factors associated with increased mortality. Methods: Our retrospective cohort study utilized the PUDF dataset and included inpatients over 60-year-old, diagnosed with kidney cancer, and hospitalized within 30-day. Person’s Chi-Square was used to measure the differences between survivors and non-survivors, and the Mann-Whitney test was for non-normality distribution for continuous variables. Then, a binary logistic regression was employed to identify the association between independent variables and mortality. Results: Five hundred and twenty-two patients were included in the study, of which 7 (1.4%) died during hospitalization. According to the univariate analysis and Mann-Whitney test, expired patients were more likely to experience older age (p = 0.005), longer length of stay (p = 0.009), ICU (p = 0.012), HMO Medicare Risk (p = 0.005), Covid-19 (p < 0.001), paralysis (p < 0.001), and higher illness severity (p < 0.001). The binary logistic regression revealed that older age (OR = 1.120, 95% CI: 1.004-1.249, p = 0.042) and the SOI (OR = 4.635, 95% CI: 1.339-16.052, p = 0.016) had significantly high odds of mortality. Conclusion: The retrospective cohort study reveals that although Covid-19 was not a predictive factor associated with increased mortality, there was a statistically significant difference between the survivor and non-survivor groups. Further studies need to assess its association with kidney cancer or other various types of cancer.

